# Who is to blame, the chicken or the egg?

**DOI:** 10.20945/2359-3997000000467

**Published:** 2022-04-28

**Authors:** Andrei C. Sposito

**Affiliations:** 1 Universidade Estadual de Campinas Campinas SP Brasil Universidade Estadual de Campinas, Unicamp, Campinas, SP, Brasil; 2 Unicamp Divisão de Cardiologia Laboratório de Aterosclerose e Biologia Vascular (Aterolab) SP Brasil Laboratório de Aterosclerose e Biologia Vascular (Aterolab), Divisão de Cardiologia, Unicamp, SP, Brasil

While there is no more room to debate the contribution of cholesterol in the development of atherosclerotic disease, the role of cholesterol-rich foods in this outcome is much less clear. In this context, the consumption of eggs has received special attention over the years, and its critical analysis has been treated in a sophisticated, precise, and objective way by Professor Eder Quintão (1) in this issue of the AE&M. Consistent with this review, a recent meta-analysis of cohort studies with up to 32 years of follow-up and over 5.54 million person-years pointed to the enormous variation in the effects obtained from egg consumption. Most subjects consumed between one and less than five eggs a week. Participants with the highest egg intake had a higher body mass index, were less likely to be treated with statins and consumed more red meat. By far, the most important finding was that in a pooled multivariate analysis, consumption of at least one egg per day was not associated with cardiovascular disease (CVD) risk after adjustment for updated lifestyle and dietary factors related with egg intake. Results were similar for coronary heart disease and stroke. In line with Eder Quintão’s comment on the predominant consumption of carbohydrates in Asians, egg consumption in this population was associated with an 8% reduction in the risk of cardiovascular events. It was not possible to clarify whether egg consumption was associated with reduced carbohydrate consumption.

Assessing the effect of diets on cardiovascular risk is almost impossible due to a set of characteristics. First, foods are composed of hundreds or thousands of molecules whose actions can diverge or add up in a pathophysiologic process. Cholesterol consumption is often associated with the ingestion of saturated fat and salt, both of which can have direct cardiovascular effects. In addition to cholesterol, eggs are composed of proteins, minerals, fatty acids, phospholipids, vitamins, and carbohydrates. The most abundant phospholipid in eggs, phosphatidylcholine, contains choline, which can be converted to trimethylamine by gut bacteria. Trimethylamine is then absorbed into the circulation and modified in the liver by the flavin-containing monooxygenase to trimethylamine N-oxide (TMAO). TMAO levels are associated with cardiovascular disease risk even after adjustment for other CVD risk factors (2).

Second, not only because of the variation in the intestinal absorption capacity of cholesterol, the effects of egg consumption on cardiovascular health may change. The absorption of the other components of eggs can also be influenced by the presence of comorbidities and dietary habits. Vegans, for example, do not produce measurable concentrations of TMAO after a high-choline diet (3). Omnivores, on the other hand, while producing TMAO in measurable amounts in the hours following consumption, are highly variable in their TMAO production and can reach levels close to zero, like vegans, up to 30 μmol/L (3). In young healthy subjects consuming up to three eggs per day for up to 4 weeks, plasma concentrations of low-density lipoprotein (HDL) were elevated and TMAO levels were unchanged, despite increases in plasma choline levels (4).

Third, unlike a medication whose use can be individualized, eating habits are part of a set of characteristics. An example of this paradigm occurred a few years ago when, in a prospective cohort, increased vitamin E intake was associated with a reduced risk of cardiovascular events (5). Subsequently, randomization for the use of vitamin E did not confirm the effect and a new interpretation of the data showed that vitamin E consumers have healthier habits (6). Increased egg consumption, for example, is associated with higher intakes of energy, protein, total fat, monounsaturated fat, and several micronutrients than non-consumers (7). Thus, even if we randomize to increase egg consumption, we will possibly be influencing other habits that can generate a confusing effect. In this context, we will be able to conclude on the safety of egg consumption in special populations, such as in familial hypercholesterolemia (FH), as commented by Dr Quintão (1), but we will have to stick to our conclusion about the consuming eggs’ habit and not to the direct outcome of this consumption.

Finally, some intriguing elements return cyclically to the debate about extrapolating population data to individual data when it comes to cardiovascular risk factors. Even monogenic diseases with high phenotypic consistency can surprise us with atypical manifestations. Among middle-aged individuals with familial hypercholesterolemia, cases without atherosclerotic cardiovascular disease are not infrequent (8). Disease manifestations result from a complex network where monogenic, polygenic, environmental, psychogenic, and socio-economic stimuli interact. Thus, although it is possible to identify the association of a certain biomarker and cardiovascular risk in population studies, it is intangible to estimate this effect when facing an individual. The use of omics and artificial intelligence (AI) has been currently the strategy under investigation to mitigate this dilemma. Data such as genomics, metabolomics, and even exposome can be obtained and added to the clinical evaluation; the latter indicating the effects of the exposure to environmental factors. The aim is to estimate the cardiovascular risk by considering the additive and attenuating effects generated by the grouping of these characteristics. In patients with acute coronary syndromes, for example, the use of this approach increases the ability to predict the risk of recurrent events, as estimated through Receiver Operating Characteristic (ROC) curve from the usual 0.7 to 0.9, with 1.0 being the maximum and 0.5 being the lowest value (9).

In conclusion, proving a causal relationship between an eating habit, such as egg consumption, and the manifestation of a disease, remains a Homeric challenge. Healthier behavior patterns should always be encouraged, but the debate on the scientific rigor of these guidelines remains fragile in their foundation and, perhaps, for that very reason, unnecessary. Future studies with large sample sizes and Bayesian factor analysis may clarify this scientific question a little more. It is even possible that tools based on omics and AI allow identifying individuals with greater susceptibility to develop diseases when exposed to certain habits. Until that happens, the medical art of closely observing the metabolic consequences in our patients remains the best option.

## References

[1] Quintão ECR (2022). Does eating eggs matter?. Arch Endocrinol Metab.

[2] Tang WH, Wang Z, Levison BS, Koeth RA, Britt EB, Fu X (2013). Intestinal microbial metabolism of phosphatidylcholine and cardiovascular risk. N Engl J Med.

[3] Miller CA, Corbin KD, da Costa KA, Zhang S, Zhao X, Galanko JA (2014). Effect of egg ingestion on trimethylamine-N-oxide production in humans: a randomized, controlled, dose-response study. Am J Clin Nutr.

[4] DiMarco DM, Missimer A, Murillo AG, Lemos BS, Malysheva OV, Caudill MA (2017). Intake of up to 3 Eggs/Day Increases HDL Cholesterol and Plasma Choline While Plasma Trimethylamine-N-oxide is Unchanged in a Healthy Population. Lipids.

[5] Stampfer MJ, Hennekens CH, Manson JE, Colditz GA, Rosner B, Willett WC (1993). Vitamin E consumption and the risk of coronary disease in women. N Engl J Med.

[6] Yusuf S, Dagenais G, Pogue J, Bosch J, Sleight P, Heart Outcomes Prevention Evaluation Study I (2000). Vitamin E supplementation and cardiovascular events in high-risk patients. N Engl J Med.

[7] Vega-Lopez S, Pignotti GA, Todd M, Keller C (2015). Egg Intake and Dietary Quality among Overweight and Obese Mexican-American Postpartum Women. Nutrients.

[8] Mszar R, Grandhi GR, Valero-Elizondo J, Virani SS, Blankstein R, Blaha M (2020). Absence of Coronary Artery Calcification in Middle-Aged Familial Hypercholesterolemia Patients Without Atherosclerotic Cardiovascular Disease. JACC Cardiovasc Imaging.

[9] de Carvalho LSF, Gioppato S, Fernandez MD, Trindade BC, Silva JCQE, Miranda RGS (2020). Machine Learning Improves the Identification of Individuals With Higher Morbidity and Avoidable Health Costs After Acute Coronary Syndromes. Value Health.

